# A comparison of seasonal influenza and novel Covid-19 vaccine intentions: A cross-sectional survey of vaccine hesitant adults in England during the 2020 pandemic

**DOI:** 10.1080/21645515.2022.2085461

**Published:** 2022-07-11

**Authors:** Vivi Antonopoulou, Louis Goffe, Carly J Meyer, Aikaterini Grimani, Fiona Graham, Jan Lecouturier, Mei Yee Tang, Paul Chadwick, Falko F Sniehotta

**Affiliations:** a NIHR Policy Research Unit in Behavioural Science, Centre for Behaviour Change, Department of Clinical, Educational, and Health Psychology, University College London, London, UK; b NIHR Policy Research Unit in Behavioural Science – Population Health Sciences Institute, Faculty of Medical Sciences, Newcastle University, Newcastle upon Tyne, UK; c NIHR Policy Research Unit in Behavioural Science – Behavioural Science Group, Warwick Business School, University of Warwick, Coventry, UK; d Department of Public Health, Preventive and Social Medicine, Center for Preventive Medicine and Digital Health Baden-Wuerttemberg, Heidelberg University, Germany

**Keywords:** Pandemic, vaccine uptake, behavioral models, vaccination, Theory of Planned Behavior, Health Belief Model

## Abstract

We compared intention to receive the seasonal influenza vaccine with a prospective coronavirus (COVID-19) vaccine among undecided or COVID-19 vaccine hesitant individuals to better understand the underlying differences and similarities in factors associated with vaccine intention. We delivered a cross-sectional online survey in October–November 2020. We included psychological constructs and sociodemographic variables informed by theory. We conducted pairwise comparisons and multiple linear regression models to explore associations between vaccine intention and psychological constructs. We recruited 1,660 participants, where 47.6% responded that they would likely receive the influenza vaccine, 31.0% that they would probably not accept the vaccination and 21.4% were unsure. In relation to the prospective COVID-19 vaccine, 39.0% responded that they would likely receive the vaccination, 23.7% that they would probably not accept the vaccination and 37.3% were unsure. *Unique factors* positively associated with COVID-19 vaccine intention were: perceived knowledge sufficiency about vaccine safety, beliefs about vaccine safety, and living in an area of low deprivation. The only *unique factor* positively associated with influenza intention was past influenza behavior. The *strongest common predictors* positively associated with intention were: favorable vaccine attitudes, the anticipated regret they may feel following infection if they were not to receive a vaccine, and the expectation from family or friends to accept the vaccine. Despite overall similarities in those factors associated with vaccination intention, we identified unique influences on intention. This additional insight will help support the planning and tailoring of future immunizations programmes for the respective viruses.

## Introduction

Since 2020, the seasonal influenza (flu) seasons have overlapped with the spread of severe acute respiratory syndrome coronavirus 2 (SARS-CoV-2) known as COVID-19. The declaration of a pandemic in March 2020 triggered an international race to produce a vaccine against COVID-19.^1^ A vaccine was considered to be the most feasible and effective approach to sustainably limit the spread of the COVID-19 virus and ultimately achieve herd immunity.^2,3^ However, herd immunity requires high levels of vaccine uptake which can be limited by factors, such as access^4^ and vaccine hesitancy.^5,6^ Vaccine hesitancy is defined as a “delay in acceptance or refusal of vaccines despite availability of vaccination services” and can occur even in conditions of high accessibility.^5,7^ Vaccine-hesitant individuals hold varying degrees of indecision about specific vaccines or may be skeptical about vaccination in general. Hesitancy usually arises when there is lack of confidence in the effectiveness and safety of the vaccine.^5,8^

Reasons for hesitancy may differ by vaccine. Low perceived utility of vaccination, negative attitudes toward influenza vaccines, and prior influenza vaccine acceptance were most frequently and consistently identified as significant barriers to influenza vaccination.^9–12^ In contrast, as COVID-19 is a novel infectious disease, concerns about the safety of a newly developed COVID-19 vaccine were raised.^1,13^ Vaccine hesitancy is not a new phenomenon but understanding why people are hesitant or undecided toward a vaccine against COVID-19 in the middle of a new and evolving pandemic is of great importance. Such information is critical to the development of public health promotion campaigns to improve vaccine uptake and limit the spread of the disease and its societal impact.^14^

### The impact of co-occurrence of seasonal influenza and COVID-19

Health-care providers have raised concerns over the possibility of a concurrent influenza epidemic in addition to the COVID-19 pandemic and have stressed the need for vaccination against each virus in order to avoid a “twindemic”.^15–18^ Both seasonal influenza and COVID-19 are infectious respiratory diseases with similar symptoms. The most at-risk groups of the population are similar across both diseases, such as those over 50 years old, people with chronic co-morbidities, and people with obesity [NHS, accessed 20 September 2021]. Vaccination coupled with personal protective behaviors are the most effective and cost-effective strategies for public health protection from both illnesses,^16–19–21^ particularly for high-risk groups to avoid hospitalization^22^ and research is underway to develop a combined vaccine.^23^

The World Health Organization (WHO) recommends annual vaccination against seasonal influenza for people 65 years old and older and for people with chronic health conditions.^2^ Although rates of seasonal influenza vaccine uptake in the UK fluctuate annually, they are , typically, below national and international targets.^24,25^ Increasing uptake of the influenza vaccine during a COVID-19 pandemic is important for two reasons. Firstly, high uptake of influenza vaccine may contribute to effective containment of COVID-19.^20,26,27^ Secondly, given that there is an increased risk of severe illness and death from co-infection with influenza and COVID-19,^17,28^ high uptake of both vaccines concurrently may limit morbidity and mortality attributable to both diseases.^20,29^

While the seasonal influenza vaccine was already in use, there was no vaccine for COVID-19 available at the time when this study was conducted (October–November 2020). Various vaccines for COVID-19 were in development though not yet approved for mass use. This stimulated much public discussion, particularly regarding vaccine safety associated to their perceived rapid development and testing.^30^ As public health authorities are considering a combined delivery program and a combined vaccine for seasonal influenza and for COVID-19 in coming years,^31^ a comparative analysis of respective vaccine attitudes is important to optimize resource management and tailor vaccine promotional messaging for each or a combined vaccine.^32^

### Supporting theoretical frameworks

Theoretical models of health behavior have been used to describe and explain vaccination intention for many diseases. The Theory of Planned Behavior (TPB) and the Health Belief Model (HBM) have been used independently and in combination to understand intention to get vaccinated.^33–38^ The TPB proposes that behavior is determined by behavioral intention and perceived control. Intention is determined by attitudes toward the behavior (favorable or unfavorable), subjective norm (beliefs about whether significant others, such as family and friends, would approve of one performing the behavior in question) and perceived behavioral control (PBC;*beliefs about* one’s ability to perform or refrain from the behavior in various circumstances).^36^ In addition to those factors postulated by the TPB, the HBM highlights the importance of perceived susceptibility (subjective assessment for personal risk of infection), perceived severity (subjective assessment for the severity of infection and its potential consequences), perceived benefits (perceived benefits of taking action), perceived barriers (perceived barriers to taking action), cues to action (triggers to engagement with health-promoting behavior), self-efficacy (perception of one’s capability to perform the behavior) and other socio-demographic factors.^37,39^

### The current study

The overall aim of this study was to examine the modifiable factors contributing to vaccine acceptance for seasonal influenza and COVID-19, exploring differences that may be important if these two vaccines are to be co-administered. More specifically, we wanted to (1) assess intention for influenza vaccine in the 2020–2021 season and for a prospective COVID-19 vaccine, and compare factors influencing intention using theory-based psychological constructs (2) identify significant predictors for each vaccine in order to draw comparisons. At the time of the study, data on COVID-19 risk infection and vaccine safety and effectiveness had not been published. Those individuals who were considering having a COVID-19 vaccine were assessing the benefits of a potential vaccine-induced immunization in the context of the COVID-19 vaccine infodemic.^40–43^ Therefore, we wanted to examine the extent to which factors may be contributing differently to the public’s willingness to be vaccinated for seasonal influenza and for COVID-19.

## Method

### Design

The study employed an online cross-sectional survey design. We commissioned YouGov, a market research company, to deliver the online survey to a population sample of residents in England. YouGov uses a point-based program to incentivize survey participation. Panel members accumulate points for completing surveys and are able to redeem these either for prize draw entry or toward a cash payment. Prior to participation, through YouGov’s platform, we informed all potential participants of the purpose of the research, the institutions involved, the study’s ethical approval, the type of data we intended to collect, and how that data would be processed. Participants had to acknowledge that completion of the questionnaire was assuming consent, but they were also able withdraw at any point during completion. The survey ran from 23 October to 4 November 2020 before any of the COVID-19 vaccines were approved for use in the UK.

### Participants

We aimed to recruit a sample population that was representative with regard to age, ethnicity, English geographical regions, and local area deprivation. Further detail on our sampling strategy is published elsewhere.^44^

### Measures

#### Questionnaire structure

The survey questionnaire began with a screening question to filter out those who stated they wanted to be vaccinated for COVID-19. They were subsequently asked no further questions and excluded from participation in the study.

The survey questionnaire included belief-based statements about vaccine intention, separate to the screening question, for both seasonal influenza and COVID-19 to allow comparisons between the two vaccines. The items included in the questionnaire were developed based on prior studies.^12–33–45–48^ All psychological constructs were drawn from the extended TPB and the HBM and novel constructs that emerged from the pandemic, inclusive of anticipated regret of not being vaccinated, vaccine knowledge, and past influenza vaccine behavior. The questionnaire consisted of the three sections: (1) COVID-19 and influenza vaccination-related attitudes and intentions captured using belief-based statements; (2) health-related behaviors; and (3) socio-demographics and socio-economic characteristics. The next three paragraphs describe the items used in each of these three sections. Full details regarding each of the psychological constructs and the belief-based statements used to capture, can be found in Table S4 in Appendix B.

#### Psychological constructs

We asked participants to indicate their level of agreement with statements about: (1) intention to accept the seasonal influenza vaccination this year (2020); and (2) intention to accept COVID-19 vaccination when one would become available to them. We assessed 11 psychological factors including vaccine attitudes, vaccine subjective norms, vaccine perceived control, and anticipated regret based on the extended TPB. Based on the HBM, we assessed perceived severity, perceived susceptibility, and perceived vaccine benefits. Other factors relevant to vaccination beliefs included perceived knowledge sufficiency about vaccine safety, beliefs about vaccine safety and side effects, trust in Government and the NHS as public health authorities considered by the public as responsible for vaccine approval and skepticism for all vaccines. Responses to all these constructs were captured using a 5-point Likert scale (1 strongly disagree to 5 strongly agree).

#### Health-related measures

We captured Body Mass Index (BMI) by asking for their self-reported height and weight measurements and their general health.^49^ We asked them if they have previously had a COVID-19 infection and whether they have been shielding (remaining at home to avoid infection) at any time during the pandemic. For the past seasonal influenza vaccination, participants were asked (1) whether or not they had accepted the influenza vaccine in the past year and (2) how often they had been getting the influenza vaccine.

#### Socio-demographic and socio-economic characteristics

Participant demographics were provided by YouGov’s panel profile information and included age, gender, ethnicity, and Index of area of Multiple Deprivation decile (IMD), a measure of neighborhood deprivation.^50^ We also asked participants if they considered themselves to be a key worker in one of the industries as defined by the Office of National Statistics^51^

### Ethics

We obtained ethical approval for the study from Newcastle University Research, Policy, Intelligence, and Ethics Committee (Reference: 4399/2020) on 18 September 2020.

### Statistical analyses

Our analysis was aligned to two key objectives: (1) to measure influenza and COVID-19 vaccine intention and identify significant psychological constructs; (2) to compare those significant predictors of vaccine intention across the two vaccines. The coefficients measuring scale internal consistency, Cronbach’s alpha and Pearson’s correlations, for each psychological construct are reported in Appendix D. Statistical analysis was performed using SPSS v28.

#### Influenza and COVID-19 vaccine intention

Differences between intention for COVID-19 vaccine versus influenza and related psychological constructs were examined using paired *t*-tests and effect size (Cohen’s *d*) was computed. We also undertook further analyses to examine intention for each vaccine across three age categories (18–49, 50–64, and 65+). Significance level for all analyses was set to *p* < .05.

For a results overview, we also performed a two-way cross-tabulated analysis between intention to accept the COVID-19 vaccination and intentions to accept influenza vaccination in winter 2020–2021 for comparisons. Intention responses of ‘strongly disagree’ and ‘disagree’ were combined, and similarly for ‘strongly agree’ and ‘agree’, to form three groups (i.e. not intending to accept, intending to accept and not sure), as these groupings aid display and interpretation of the results.

#### Predictors of intention across the two vaccines

Multiple linear regression models were carried out to explore the association between vaccine intention and psychological constructs, controlling for participant demographics including age, gender, ethnicity, IMD, whether they had been infected with COVID-19 and whether they had been shielding, general health status, BMI, and keyworker status. Additionally, seasonal influenza vaccination history was also captured with two items, one on past vaccination and another one on frequency of past influenza vaccination. The main outcome variable was vaccine intention for each vaccine, respectively. First, we examined univariate associations between all key constructs (11 psychological factors, four personal health characteristics and four socio-demographic and socio-economic factors) and vaccine intention using a series of univariate linear regression models. Significant variables (*p* < .10) were considered for inclusion in the final model. Pairwise correlations were computed between all constructs and vaccine intention and all correlations estimates for each model were found to be less than 0.7, with the exception of one variable, the variable ‘attitudes’ for the seasonal influenza, which was 0.7. Subsequently, a multiple regression model with backward selection method was fitted to the data removing variables until all variables have a *p*-value equal to or less than 0.05. Multicollinearity and outliers were checked. All variation inflation factor (VIF) values were less than 3 in each model and the values of the Durbin-Watson statistics were close to 2 (1.977 for influenza and 1.893 for COVID-19). R^2^ values were reported to indicate goodness-of-fit.

## Results

### Sample characteristics

Our final sample included 1,660 participants—summary statistics on the demographic and health characteristics are presented in Table 1.

**Table 1.** Participant characteristics.

Characteristic	Level	N (%)
Gender	Female	946 (57.0%)
	Male	714 (43.0%)
Age	Under 50	1122 (67.6%)
	50-64	332 (20.0%)
	65 and over	206 (12.4%)
Ethnicity	**White**	**1297 (78.1%)**
	English/Welsh/Scottish/Northern Irish/British	1209 (72.8%)
	Irish	17 (1.0%)
	Any other White background	71 (4.3%)
	**Mixed/multiple ethnic groups**	**83 (5.0%)**
	White and Black Caribbean	20 (1.2%)
	White and Black African	7 (0.4%)
	White and Asian	31 (1.9%)
	Any other Mixed/Multiple ethnic background	25 (1.5%)
	**Asian/Asian British**	**180 (10.8%)**
	Indian	69 (4.2%)
	Pakistani	36 (2.2%)
	Bangladeshi	25 (1.5%)
	Chinese	26 (1.6%)
	Any other Asian background	24 (1.4%)
	**Black/African/Caribbean/Black British**	**88** (**5.3%)**
	African	45 (2.7%)
	Caribbean	31 (1.9%)
	Any other Black/African/Caribbean background	12 (0.7%)
	**Other ethnic group**	**12** (**0.7%)**
	Arab	3 (0.2%)
	Any other ethnic group	9 (0.5%)
England region	London	286 (17.2%)
	East Midlands	141 (8.5%)
	East of England	155 (9.3%)
	North East	67 (4.0%)
	North West	198 (11.9%)
	South East	281 (16.9%)
	South West	163 (9.8%)
	West Midlands	179 (10.8%)
	Yorkshire and the Humber	190 (11.4%)
Deciles of Indices of Multiple Deprivation	1 (most deprived)	137 (8.3%)
	2	170 (10.2%)
	3	146 (8.8%)
	4	157 (9.5%)
	5	166 (10.0%)
	6	145 (8.7%)
	7	172 (10.4%)
	8	173 (10.4%)
	9	187 (11.3%)
	10	204 (12.3%)
	Missing	3 (0.2%)
Key worker	Not a key worker	585 (35.2%)
	Not sure	103 (6.2%)
	Key worker—Health and social care	110 (6.6%)
	Key worker—Education and childcare	129 (7.8%)
	Key worker—Utilities and communication	28 (1.7%)
	Key worker—Food and necessary goods	70 (4.2%)
	Key worker—Transport	37 (2.2%)
	Key worker—Key public services	48 (2.9%)
	Key worker—Public safety and national security	15 (0.9%)
	Key worker—National and local governments	34 (2.0%)
	Not in work	501 (30.2%)
BMI	Underweight	67 (4.0%)
	Healthy weight	646 (38.9%)
	Overweight	469 (28.3%)
	Obese	283 (17.0%)
	Missing	195 (11.7%)
Previously had COVID-19	Not had COVID-19	1336 (80.5%)
	Had COVID-19	284 (17.1%)
	Missing	40 (2.4%)
Shielding from COVID-19	Yes	320 (19.3%)
	No	1340 (80.7%)
General health	Very bad	13 (0.8%)
	Bad	60 (3.6%)
	Fair	390 (23.5%)
	Good	773 (46.6%)
	Very good	358 (21.6%)
	Missing	66 (4.0%)
Seasonal influenza vaccine frequency	Never	794 (47.8%)
	Rarely	275 (16.6%)
	Some years	145 (8.7%)
	Most years	140 (8.4%)
	Every year	306 (18.4%)

### Vaccine type and intention

#### Seasonal influenza intention

Of the 1,660 participants, 47.6% (n = 790) participants responded that they would probably receive the influenza vaccine, whereas 31.0% (n = 515) reported they probably would not accept the vaccine and 21.4% (n = 355) were unsure.

#### COVID-19 intention

Of the 1,660 participants, 39.0% (n = 647) participants responded that they would probably receive the COVID-19 vaccine, whereas 23.7% (n = 394) reported they probably would not accept the vaccine and 37.3% (n = 619) were unsure.

#### Vaccine intention—cross-tabulation of vaccine intention as intersection of responses between the two vaccines

Of the 1,660 participants, 17.7% (n = 295) responded that neither intended to accept the influenza vaccine nor the COVID-19 vaccine; 28.8% (n = 478) responded that intended to accept both the influenza vaccine and the COVID-19 vaccine; 14.2% (n = 236) were undecided for both vaccines. Table 2 below presents the vaccine intention for COVID-19 and for seasonal influenza in cross-tabulation.

**Table 2.** Cross-Tabulation depicting proportion of participants’ (frequencies) indicating dis/agreement with COVID-19 vaccine intention and influenza vaccine intention (N = 1,660).

		Influenza vaccine Intention			Total Covid-19 vaccine intention
		Unwilling to accept the vaccine	Unsure	Likely to accept	
COVID-19 vaccine intention	Unwilling to accept the vaccine	295 (17.7%)	40 (2.4%)	59 (15%)	394
					**23.7%**
	Unsure	130 (7.8%)	236 (14.22%)	253 (31.5%)	619
					**37.3%**
	Likely to accept	90 (13.9%)	79 (4.75%)	478 (28.8%)	647
					**39.0%**
Total Influenza vaccine intention		515	355	790	1660
		**31.1%**	**21.4%**	**47.6%**	100.0%

### Vaccine type, vaccination intention, and psychological constructs

The mean values and standard deviation (SD) for participants’ responses to questionnaire items about the seasonal influenza vaccine and their responses about a prospective COVID-19 vaccine are shown in Table 3. The mean response for influenza intention was M = 3.27 (SD = 1.33) compared with the mean response for COVID-19 vaccine which was M = 3.11 (SD = 1.07) and this difference was statistically significant (t(1,659) = 5.74, *p* < .001), although the effect size shows a small effect (0.14).

**Table 3.** Mean scores and pairwise comparisons for Influenza and COVID-19 responses (N = 1,660).

Construct	Seasonal Influenza	COVID-19-19	T-test	Effect Size, Cohen’s *d*
	Mean (SD)	Mean (SD)		
Intention	3.27 (1.33)	3.11 (1.07)	t(1,659) = 5.74, *p* < .001	*d* = 0.14
Vaccine Attitudes	3.81 (1.09)	3.76 (1.10)	t(1,659)= −2.29, *p* = .022	*d* = 0.06
Subjective Norms	2.62 (0.97)	3.16 (0.89)	t(1,659)= −22.47, *p* < .001	*d* = 0.05
Perceived Severity	2.64 (1.02)	3.01 (1.03)	t(1,659)= −19.104, *p* < .001	*d* = 0.51
Anticipated regret	3.28 (1.27)	3.55 (1.30)	t(1,659)= −12.61, *p* < .001	*d* = 0.31
Knowledge sufficiency	3.56 (1.03)	2.56 (1.11)	t(1,659) = 31.95, *p* < .001	*d* = 0.78
Vaccine benefits	2.90 (0.70)	2.93 (0.75)	t(1,659)= −1.30, *p* > .05	*d* = 0.03
Vaccine safety/side effects	3.69 (0.87)	3.11 (0.84)	t(1,659) = 29.86, *p* < .001	*d* = 0.73
Trust to Authorities	3.64 (0.93)	3.16 (0.97)	t(1659) = 24.56, *p* < .001	*d=*0.60
Perceived control	4.10 (0.99)	3.45 (1.11)	t(1,659) = 22.32, *p* < .001	*d* = 0.55
Susceptibility	2.44 (1.09)	2.55 (1.11)	t(1,659)= −4.521, *p* < .001	*d* = 0.11
*Sub-analyses*				
**Vaccine perceived safety (per each component):**				
Mild vaccine side effects	3.61 (0.95)	3.02 (0.85)	t(1,659) = 25.83, *p* < .001	*d* = 0.63
Vaccine fully tested	3.77 (0.96)	3.22 (1.01)	t(1659) = 25.57, *p* < .001	*d* = 0.63
**Attitudes (per each component):**				
Worthless/Valuable	3.61 (1.33)	3.71 (1.25)	t(1,659) = 3.48, *p* < .001	*d* = 0.09
Harmful/Beneficial	3.79 (1.18)	3.73 (1.23)	t(1,659)= −2.48, *p* < .05	*d* = 0.06
Painful/Tolerable	4.03 (1.14)	3.84 (1.18)	t(1,659)= −7.76, *p* < .001	*d* = 0.19

Statistically significant differences between the influenza and the COVID-19 vaccine questions were found for all the psychological constructs with the exception of vaccine perceived benefits, which did not yield a significant difference (*p* > .05). The means for the vaccine attitudes, knowledge sufficiency, safety, trust in government and NHS approved vaccine, perceived control and mild side effects from the vaccine were higher for the seasonal influenza vaccine. In contrast, the means for subjective norms, perceived severity, anticipated regret, and perceived susceptibility were higher for the COVID-19 vaccine and these differences between the two vaccines were found to be significantly different, although the effect sizes indicate very small effects—with the exception of knowledge sufficiency which yield a large effect (approx. 0.8) according to Cohen.^52^

#### Vaccine type, vaccination intention, and psychological constructs: comparisons by age categories

Comparative analyses conducted by age category (i.e. age categories 18–49, 50–64 and 65+) revealed higher intention for the influenza vaccine across all age groups, although this difference did not reach statistical significance for the age category 18–49. In contrast, a statistically significant difference was found both for the age group category 50–64 and similarly for the age group category of over 65+ in terms of vaccine intention. The effect size of the statical difference between reported intention for influenza and for COVID-19 in the age category 50–64 was small (d = 0.19) but it was considerable in the age group category 65+ (d = 0.64) according to Cohen.^52^ Table 4 below presents the means and paired *t*-tests for vaccine intention per age categories.

**Table 4.** Influenza versus COVID-19 vaccine intention pairwise comparisons per age groups.

Intention	N	Seasonal Influenza	COVID-19	T-test	Effect Size, Cohen’s *d*
		Mean (SD)	Mean (SD)		
Age group 18-49	(n = 1,122)	3.13 (1.25)	3.08 (1.05)	t(1,121) = 1.44, *p* > .05	*d* = 0.04
Age group 50-64	(n = 332)	3.27 (1.09)	3.04 (1.12)	t(331) = 3.54, *p* < .001	*d* = 0.19
Age group 65+	(n = 206)	4.03 (1.39)	3.35 (1.09)	t(205) = 9.19, *p* < .001	*d* = 0.64

Statistically significant differences were shown across all influenza and COVID-19 psychological constructs for all age group categories with the exception of the following pairwise comparisons: vaccine attitudes (t(1,121) = 0.079, *p* = .93) for the age group 18–49; vaccine attitudes (t(331) = 3.538, *p* = .06) and vaccine benefits (t(331) = 0.001, *p* = 1.00) for the age group category 50–64; and subjective norms (t(205) = 0.641, *p* = .52)). All pairwise comparisons per age category are displayed in Table S1 in Appendix A.

### Significant predictors for influenza vaccine intention versus COVID-19 vaccine intention

#### Predictors for influenza vaccine intention

Backward multiple regression was conducted to identify a parsimonious combination of psychological constructs and health and sociodemographic variables as predictors for influenza vaccine intention. The model was significant (F(8,1395) = 371.530, *p* < .001) and was based on 1,395 participants and revealed 8 significant predictors. These factors included five psychological constructs: vaccine attitudes, subjective norms, anticipated regret, vaccine benefits, and trust to authorities approving vaccines, and two socio-demographic and health-related factors: being a health-care worker and frequency of past influenza vaccination. All factors were positively associated with influenza vaccine intention with the exception of being a health-care worker, which was negatively associated. The beta weighs and significance and VIF values for the final model are presented in Table 5. The adjusted R^2^ was 0.678 which is a large effect^52^ signifying that a high proportion of the variance (68%) was explained by the model.

**Table 5.** Multiple regression estimates for seasonal influenza vaccine intention, displaying coefficients [95% CIs] and VIF coefficients (N = 1,660).

	Seasonal Influenza vaccine responses		95% Confidence Interval for B		
Construct	b	*p*-value	Lower Bound	Upper Bound	VIF
Vaccine Attitudes	0.302	<.001	0.323	0.424	1.894
Subjective Norm	0.169	<.001	0.179	0.278	1.541
Anticipated Regret	0.193	<.001	0.162	0.247	1.800
Vaccine benefits	0.074	<.001	0.071	0.214	1.548
Trust to Authorities for vaccine approval	0.103	<.001	0.093	0.202	1.622
Frequency of past influenza vaccination	0.276	<.001	0.200	0.265	1.688
Healthcare worker status	−0.033	.033	−0.167	−0.007	1.017

Adjusted R^2^ for influenza vaccine intention: 67.8.

#### Predictors for COVID-19 vaccine intention

The regression model that we fit to the COVID-19 data was also significant (F(11,1392) = 190.020, *p* < .001, n = 1,392) and revealed 11 significant predictors. These factors included seven psychological constructs: vaccine attitudes, subjective norms, anticipated regret, knowledge sufficiency, vaccine benefits, vaccine safety, and trust in authorities approving vaccines; one heath characteristic: personal health status; two socio-demographic factors: index of area deprivation and being a health-care worker, and past influenza vaccination. All factors were positively associated with influenza vaccine intention with the exception of personal health status, and being a health-care worker, which were both negatively associated. The beta weighs and significance and VIF values for the final model are presented in Table 6. The adjusted R^2^ was 0.597 which is a large effect^52^ and signifies that a high proportion of the variance (60%) was explained by the model.

**Table 6.** Multiple regression estimates for COVID-19 vaccine intention, displaying coefficients [95% CIs] and VIF coefficients (N = 1,660).

	COVID-19 vaccine responses		95% Confidence Interval for B		
Construct	b	*p*-value	Lower Bound	Upper Bound	VIF
Vaccine Attitudes	0.242	<.001	0.194	0.288	2.037
Subjective Norm	0.190	<.001	0.183	0.276	1.361
Anticipated Regret	0.188	<.001	0.119	0.196	1.921
Vaccine knowledge sufficiency	0.047	.008	0.012	0.079	1.077
Vaccine benefits	0.128	<.001	0.117	0.253	2.018
Perceived vaccine safety	0.069	.013	0.019	0.161	2.693
Trust to Authorities for vaccine approval	0.171	<.001	0.127	0.253	2.912
Past influenza vaccine behavior	0.037	.030	0.001	0.006	1.019
Personal health status	−0.037	.033	−0.094	−0.004	1.056
Index of Multiple Deprivation	0.035	.040	0.001	0.026	1.030
Healthcare worker status	−0.036	.036	−0.149	−0.005	1.009

Adjusted R^2^ for COVID-19 vaccine intention: 59.7.

## Discussion

In this study, we explored factors associated with the intention to receive influenza and COVID-19 vaccinations in individuals in England at the height of the pandemic. Willingness to be vaccinated against the seasonal influenza was significantly higher than for COVID-19, particularly in those aged 50 and over, although the overall difference was small in terms of effect size. In terms of predictors, most of the factors that were associated with intention to vaccinate were similar for the two vaccines, but some unique factors predicting intention were found for each vaccine.

### Unique influences on vaccination intention: Covid-19

Intention to receive a vaccine for COVID-19 was uniquely positively associated with perceived knowledge sufficiency about vaccine safety, beliefs about the vaccine safety, and previous influenza vaccination behavior. People were more likely to accept the COVID-19 vaccine if they thought they had sufficient information to make an informed decision about vaccine safety, if they believed the vaccine would be safe with either none or mild side effects, and if they had accepted the influenza vaccine the previous year and if they lived in more affluent areas. In contrast, people were less likely to accept the COVID-19 vaccine if they were in good health.

The significant predictors of vaccine safety and vaccine knowledge for COVID-19 vaccine intention indicates that fear and safety concerns for a novel vaccine outweigh the perceived risks associated with COVID-19 infection. Other studies have also found concerns about adverse effects and vaccine novelty to be associated with COVID-19 vaccination intention.^45,53^ Area deprivation as a predictor of intention aligns with previous work showing lower levels of actual vaccination in more deprived areas,^54,55^ which has been linked to lower levels of trust in government and public health authorities.^55^ In our analysis, socio-economic status and trust were both independently associated with vaccination intention, suggesting that the influence of socio-economic status extends beyond its association with trust.

### Unique influences on vaccination intention: influenza

In relation to influenza vaccine intention, the unique factor positively associated with vaccine intention was frequency of past influenza vaccination. People were more likely to receive the influenza vaccine if they had frequently received a vaccine in the past. This finding aligns to related studies.^45,56^

### Comparisons of the different factors in vaccine intention between vaccines

Our study identified critical differences in relation to vaccine intention between the two vaccines: knowledge of vaccine safety and perceived vaccine safety. Despite responses about perceived severity and perceived susceptibility denoting acknowledgment of higher risk for COVID-19 among our sample, there was still an increased acceptance of influenza vaccination, in comparison to the COVID-19 vaccine. Thus, these results denote that lack of confidence in the safety of vaccines outweighed the perceived disease risks even during a pandemic when it is so critical for an individual to decide whether or not to accept the vaccine. However, it is important to note that this sample was selected based on their COVID-19 vaccine intention. Overall, these findings are in line with recent studies showing that the novelty of the vaccine and its safety and effectiveness, possible vaccine side effects, and severity of side effects are reported as negative influences on the decision to vaccinate against COVID-19.^57–62^ The development and approval of the COVID-19 vaccine has indeed occurred at an unprecedented speed with some voicing concerns about unknown side-effects.^61,63^ Therefore, transparency in how a vaccine is being developed and clarifications regarding vaccine side-effects is highly critical.^64^

### Common factors affecting vaccination intention for both vaccines

We also identified many similarities in the profile of factors affecting vaccination intention. Previous vaccination behavior, favorable attitudes toward vaccination, perceived benefits from vaccination, trust in authorities associated with vaccine approval, perceived regret from not having accepted the vaccine and infecting others, as well as the belief that significant others around them expect them to accept a COVID-19 and influenza vaccine were identified as factors predicting vaccination intention for both vaccines. These findings are in line with similar research on vaccine intention for influenza^27,45,65^ and for COVID-19.^34,59,66,67^

Being a health-care professional was negatively associated with the influenza vaccine intention which indicates lower intention. Low levels of vaccine acceptance among health-care professionals has been widely reported in other studies for the influenza vaccine^68–70^ and for COVID-19 vaccine.^71–73^ Interestingly, age was not found to be a significant predictor for either vaccine.

### Policy implications

Our finding of unique predictors but also commonalities in the psychosocial factors associated with vaccination intention across these two diseases has implications for the planning of future vaccination programs, particularly those that aim to vaccinate against both diseases simultaneously.

With the ongoing circulation of new variants, this suggests that repeated vaccination for COVID-19 will likely be necessary. The parallels with seasonal influenza vaccination can be drawn to promote vaccination uptake in both the high-risk groups and the general population,^45,46,54,56,67,74^ particularly as there is evidence to suggest that there is a significant decrease in risk of COVID-19 infection after receiving the influenza vaccine.^75^ Public health authorities must be vigilant regarding the public’s concerns regarding vaccine effectiveness against new variants and the modification of existing vaccines, as these factors can undermine trust in vaccines.^76,77^ Although the majority of individuals in England are favorable toward vaccination, concerted efforts by a small number of individuals can undermine trust in an immunization program. Therefore, it is important that public health authorities are quick to address vaccine safety and efficacy concerns.^78,79^ Tailored health messaging campaigns are cost-effective and can change people’s attitudes and behaviors leading to an informed health promoting decision. A recent study conducted in the UK on information provision about the safety and efficacy of the new COVID-19 vaccines resulted in vaccination intentions that were, on average, 0.39 standard deviations (SDs) higher than those in the ‘no information’ condition.^32^ Interestingly, researchers found that providing the same COVID-19 vaccine efficacy information in the context of information about seasonal influenza vaccine efficacy resulted in a further significant increase in vaccination intentions that were 0.68 SD higher than those in the ‘no information’ condition without undermining influenza vaccine intentions. This is supported by our findings where we identified past influenza vaccine behavior as a significant predictor both for COVID-19 and for influenza intention. Influenza vaccination is a cost-effective way in reducing influenza-like illnesses and therefore, can be utilized as a vaccination familiarization factor for reducing vaccine hesitancy and promote a pro-vaccine culture.^80–82^ This finding is encouraging from a public health perspective. It means that protocols and lessons learned from previous efforts to increase seasonal influenza vaccination are relevant to the design and delivery of future public health interventions.^80^ This is particularly relevant in the context of the decision by the Joint Committee on Vaccination and Immunization to change the eligibility criteria of the seasonal influenza NHS vaccination program for 2022/23 in line with pre-pandemic recommendations.^83^

### Limitations of the study

Our study was limited in that we measured self-reported vaccination intentions rather than actual vaccination behavior and the intention-behavior gap is widely established.^84^ However, as the COVID-19 vaccine was not yet available, this was not possible. Moreover, this was an online cross-sectional study and while this method is useful for rapid collection of data, this method introduces biases in the data in terms of sampling representativeness: requiring internet access, being registered with the survey platform and the temporal aspects of data collection which reflects vaccination intention at the specific time point of data collection. Whilst we achieved a sizable number of older survey participants in comparison to related studies,^46,67^ it should be noted that the overall proportion of those aged 65 and over was only 12.4%. This age group is comprised of the highest proportion of internet non-users and are therefore the most challenging age group to recruit for online-only surveys.^85^ It is also important to note that this was a COVID-19 hesitant sample but not necessarily an influenza hesitant sample and hence, some differences in the predictive power of the models may be due to this selection criterion. In hindsight, an additional limitation in relation to the predictive power of these models and the policy implications should be acknowledged; the availability of different COVID-19 vaccines approved shortly after the study was conducted and the huge amount of contradictory information (infodemics) may have influenced people’s attitudes and behaviors toward COVID-19 and influenza vaccines, with consequent impact on communication strategies.^86^

### Future research

Several additional factors could be explored to improve our models of vaccine intention including systems-level factors which have been found to impact seasonal influenza vaccine and COVID-19 vaccine acceptance.^86–88^ Access barriers, including location and time of vaccine delivery, are factors related to the provision of health services and may have increased the disparities in reported intention and uptake.^89^ Future research needs to examine issues relating to vaccine delivery such as access to preventative care services, timing, availability and location of appointments for vaccinations, and vaccination experience in order to assess the degree to which these factors impact willingness to accept vaccination. The contribution of these factors on uptake intention of the COVID-19 vaccination booster program (2021–2022) in the UK has been examined by the authors in another study (Meyer et al., under review).

## Conclusion

The success of the vaccine rollout program is dependent on the effectiveness of the vaccines against the virus (and variants) and population uptake, both of which need to be constantly monitored. Our study contributes to an increased understanding of the key factors underlying vaccine intentions and can help inform policy recommendations for the current vaccination program against COVID-19 in England. It is also supportive of future immunization strategies, particularly given the consideration of a jointly administered influenza and COVID-19 vaccination program. Public health authorities could potentially increase seasonal influenza and COVID-19 vaccine uptake through education and outreach by focusing on addressing vaccine safety and efficacy and by highlighting perceived benefits of vaccination for specific groups including health-care professionals and for communities in areas of high deprivation.

## Supplementary Material

Supplemental Material

## Data Availability

The data presented in this study are available upon reasonable request from the corresponding author.
