# Supplementary material for: A comparison of seasonal influenza and novel Covid-19 vaccine intentions: A cross-sectional survey of vaccine hesitant adults in England during the 2020 pandemic
Source: Hum Vaccin Immunother. 2022 Jul 11;18(5):2085461. doi: 10.1080/21645515.2022.2085461 (PMC9621000; doi:10.1080/21645515.2022.2085461)
Supplement: Supplemental Material [file KHVI_A_2085461_SM5629.docx]

**Appendix A**

*Supplementary tables*

**Table S1.** Pairwise comparisons for influenza versus COVID-19 vaccine intention by age categories

| **Age categories** | | | **Constructs** | **Mean** | | | **N** | **Std. Deviation** | **Std. Error Mean** | | **t-test** | | **df** | | **p-value** | | **Effect size, Cohen’s *d*** | |
| --- | --- | --- | --- | --- | --- | --- | --- | --- | --- | --- | --- | --- | --- | --- | --- | --- | --- | --- |
| **18-49** | Pair 1 | Influenza vaccine intention | | | 3.13 | 1122 | | 1.26 | 0.04 | 1.441 | | 1121 | | 0.15 | | 0.04 | |  |
|  |  | Covid-19 vaccine intention | | | 3.08 | 1122 | | 1.05 | 0.03 |  | |  | |  | |  | |  |
|  | Pair 2 | Attitudes for influenza | | | 3.73 | 1122 | | 1.05 | 0.03 | 0.079 | | 1121 | | 0.937 | | 0.00 | |  |
|  |  | Attitudes for COVID-19 | | | 3.73 | 1122 | | 1.11 | 0.03 |  | |  | |  | |  | |  |
|  | Pair 3 | Subjective Norm for influenza | | | 2.43 | 1122 | | 0.94 | 0.03 | -23.231 | | 1121 | | <.001 | | -0.69 | |  |
|  |  | Subjective Norm for COVID-19 | | | 3.14 | 1122 | | 0.90 | 0.03 |  | |  | |  | |  | |  |
|  | Pair 4 | Perceived severity for influenza | | | 2.44 | 1122 | | 0.98 | 0.03 | -15.853 | | 1121 | | <.001 | | -0.47 | |  |
|  |  | Perceived severity for COVID-19 | | | 2.82 | 1122 | | 1.01 | 0.03 |  | |  | |  | |  | |  |
|  | Pair 5 | Anticipated Regret for influenza | | | 3.20 | 1122 | | 1.24 | 0.04 | -10.036 | | 1121 | | <.001 | | -0.30 | |  |
|  |  | Anticipated Regret for COVID-19 | | | 3.47 | 1122 | | 1.29 | 0.04 |  | |  | |  | |  | |  |
|  | Pair 6 | Perceived influenza vaccine knowledge sufficiency | | | 3.47 | 1122 | | 1.04 | 0.03 | 24.414 | | 1121 | | <.001 | | 0.73 | |  |
|  |  | Perceived COVID-19 vaccine knowledge sufficiency | | | 2.51 | 1122 | | 1.11 | 0.03 |  | |  | |  | |  | |  |
|  | Pair 7 | Influenza vaccine benefits | | | 2.90 | 1122 | | 0.70 | 0.02 | -2.685 | | 1121 | | 0.007 | | -0.08 | |  |
|  |  | COVID-19 vaccine benefits | | | 2.94 | 1122 | | 0.78 | 0.02 |  | |  | |  | |  | |  |
|  | Pair 8 | Influenza vaccine safety | | | 3.68 | 1122 | | 0.88 | 0.03 | 23.656 | | 1121 | | <.001 | | 0.71 | |  |
|  |  | COVID-19 vaccine safety | | | 3.13 | 1122 | | 0.88 | 0.03 |  | |  | |  | |  | |  |
|  | Pair 9 | Trust to Authorities for influenza vaccine approval | | | 3.62 | 1122 | | 0.94 | 0.03 | 19.689 | | 1121 | | <.001 | | 0.59 | |  |
|  |  | Trust to Authorities for COVID-19 vaccine approval | | | 3.15 | 1122 | | 0.99 | 0.03 |  | |  | |  | |  | |  |
|  | Pair 10 | Perceived control for influenza | | | 4.00 | 1122 | | 1.02 | 0.03 | 19.027 | | 1121 | | <.001 | | 0.57 | |  |
|  |  | Perceived control for COVID-19 | | | 3.30 | 1122 | | 1.11 | 0.03 |  | |  | |  | |  | |  |
|  | Pair 11 | Perceived susceptibility to influenza  Perceived susceptibility to COVID-19 | | | 2.31  2.42 | 1122  1122 | | 1.08  1.09 | 0.03  0.03 | -4.093 | | 1121 | | <.001 | | -0.12 | |  |
| **50-64** | Pair 1 | Influenza vaccine intention | | | 3.27 | 332 | | 1.36 | 0.07 | 3.538 | | 331 | | <.001 | | 0.19 | |  |
|  |  | Covid-19 vaccine intention | | | 3.04 | 332 | | 1.12 | 0.06 |  | |  | |  | |  | |  |
|  | Pair 2 | Attitudes for influenza | | | 3.81 | 332 | | 1.14 | 0.06 | 1.871 | | 331 | | 0.062 | | 0.10 | |  |
|  |  | Attitudes for COVID-19 | | | 3.71 | 332 | | 1.10 | 0.06 |  | |  | |  | |  | |  |
|  | Pair 3 | Subjective Norm for influenza | | | 2.76 | 332 | | 0.92 | 0.05 | -7.626 | | 331 | | <.001 | | -0.42 | |  |
|  |  | Subjective Norm for COVID-19 | | | 3.11 | 332 | | 0.91 | 0.05 |  | |  | |  | |  | |  |
|  | Pair 4 | Perceived severity for influenza | | | 2.86 | 332 | | 0.97 | 0.05 | -8.086 | | 331 | | <.001 | | -0.44 | |  |
|  |  | Perceived severity for COVID-19 | | | 3.19 | 332 | | 0.93 | 0.05 |  | |  | |  | |  | |  |
|  | Pair 5 | Anticipated Regret for influenza | | | 3.18 | 332 | | 1.27 | 0.07 | -7.91 | | 331 | | <.001 | | -0.43 | |  |
|  |  | Anticipated Regret for COVID-19 | | | 3.52 | 332 | | 1.30 | 0.07 |  | |  | |  | |  | |  |
|  | Pair 6 | Perceived influenza vaccine knowledge sufficiency | | | 3.59 | 332 | | 1.04 | 0.06 | 15.596 | | 331 | | <.001 | | 0.86 | |  |
|  |  | Perceived COVID-19 vaccine knowledge sufficiency | | | 2.57 | 332 | | 1.12 | 0.06 |  | |  | |  | |  | |  |
|  | Pair 7 | Influenza vaccine benefits | | | 2.84 | 332 | | 0.68 | 0.04 | 0.001 | | 331 | | 1.00 | | 0.00 | |  |
|  |  | COVID-19 vaccine benefits | | | 2.84 | 332 | | 0.71 | 0.04 |  | |  | |  | |  | |  |
|  | Pair 8 | Influenza vaccine safety | | | 3.64 | 332 | | 0.88 | 0.05 | 13.887 | | 331 | | <.001 | | 0.76 | |  |
|  |  | COVID-19 vaccine safety | | | 3.03 | 332 | | 0.79 | 0.04 |  | |  | |  | |  | |  |
|  | Pair 9 | Trust to Authorities for influenza vaccine approval | | | 3.57 | 332 | | 0.94 | 0.05 | 11.496 | | 331 | | <.001 | | 0.63 | |  |
|  |  | Trust to Authorities for COVID-19 vaccine approval | | | 3.05 | 332 | | 0.97 | 0.05 |  | |  | |  | |  | |  |
|  | Pair 10 | Perceived control for influenza | | | 4.21 | 332 | | 0.94 | 0.05 | 8.979 | | 331 | | <.001 | | 0.49 | |  |
|  |  | Perceived control for COVID-19 | | | 3.65 | 332 | | 1.06 | 0.06 |  | |  | |  | |  | |  |
|  | Pair 11 | Perceived susceptibility to influenza  Perceived susceptibility to COVID-19 | | | 2.55  2.60 | 332  332 | | 1.05  1.04 | 0.05  0.06 | -0.792 | | 331 | | <0.05 | | -0.043 | |  |
|  |  |  | | |  |  | |  |  |  | |  | |  | |  | |  |
| **65+** | Pair 1 | Influenza vaccine intention | | | 4.03 | 206 | | 1.39 | 0.10 | 9.192 | | 205 | | <.001 | | 0.64 | |  |
|  |  | Covid-19 vaccine intention | | | 3.35 | 206 | | 1.09 | 0.08 |  | |  | |  | |  | |  |
|  | Pair 2 | Attitudes for influenza | | | 4.24 | 206 | | 1.08 | 0.08 | 3.634 | | 205 | | <.001 | | 0.25 | |  |
|  |  | Attitudes for COVID-19 | | | 4.00 | 206 | | 1.07 | 0.07 |  | |  | |  | |  | |  |
|  | Pair 3 | Subjective Norm for influenza | | | 3.40 | 206 | | 0.83 | 0.06 | 0.641 | | 205 | | 0.523 | | 0.05 | |  |
|  |  | Subjective Norm for COVID-19 | | | 3.37 | 206 | | 0.81 | 0.06 |  | |  | |  | |  | |  |
|  | Pair 4 | Perceived severity for influenza | | | 3.38 | 206 | | 0.94 | 0.07 | -7.057 | | 205 | | <.001 | | -0.49 | |  |
|  |  | Perceived severity for COVID-19 | | | 3.71 | 206 | | 0.91 | 0.06 |  | |  | |  | |  | |  |
|  | Pair 5 | Anticipated Regret for influenza | | | 3.88 | 206 | | 1.25 | 0.09 | -2.448 | | 205 | | 0.015 | | -0.17 | |  |
|  |  | Anticipated Regret for COVID-19 | | | 4.00 | 206 | | 1.25 | 0.09 |  | |  | |  | |  | |  |
|  | Pair 6 | Perceived influenza vaccine knowledge sufficiency | | | 4.02 | 206 | | 0.83 | 0.06 | 14.574 | | 205 | | <.001 | | 1.02 | |  |
|  |  | Perceived COVID-19 vaccine knowledge sufficiency | | | 2.80 | 206 | | 1.08 | 0.08 |  | |  | |  | |  | |  |
|  | Pair 7 | Influenza vaccine benefits | | | 3.07 | 206 | | 0.73 | 0.05 | 3.098 | | 205 | | 0.002 | | 0.22 | |  |
|  |  | COVID-19 vaccine benefits | | | 2.97 | 206 | | 0.64 | 0.04 |  | |  | |  | |  | |  |
|  | Pair 8 | Influenza vaccine safety | | | 3.82 | 206 | | 0.87 | 0.06 | 12.22 | | 205 | | <.001 | | 0.85 | |  |
|  |  | COVID-19 vaccine safety | | | 3.23 | 206 | | 0.70 | 0.05 |  | |  | |  | |  | |  |
|  | Pair 9 | Trust to Authorities for influenza vaccine approval | | | 3.86 | 206 | | 0.85 | 0.06 | 9.303 | | 205 | | <.001 | | 0.65 | |  |
|  |  | Trust to Authorities for COVID-19 vaccine approval | | | 3.40 | 206 | | 0.82 | 0.06 |  | |  | |  | |  | |  |
|  | Pair 10 | Perceived control for influenza | | | 4.48 | 206 | | 0.81 | 0.06 | 7.675 | | 205 | | <.001 | | 0.54 | |  |
|  |  | Perceived control for COVID-19 | | | 3.96 | 206 | | 0.95 | 0.07 |  | |  | |  | |  | |  |
|  | Pair 11 | Perceived susceptibility to influenza  Perceived susceptibility to COVID-19 | | | 2.99  3.15 | 206  206 | | 1.06  1.10 | 0.08  0.07 | -2.262 | | 205 | | p=0.025 | | -0.16 | |  |
|  | |  | | |  |  | |  |  |  | |  | |  | |  | |  |
|  |  |  | | |  |  | |  |  |  | |  | |  | |  | |  |

**Table S2**. Full model for influenza and for COVID-19 vaccine: multiple linear regression estimates for Influenza and for COVID-19 vaccine intention, displaying coefficients [95% CIs] and VIF coefficients (N=1,660)

|  | **Seasonal Influenza vaccine responses** |  | 95% Confidence Interval for B | |  | **COVID-19 vaccine responses** |  | 95% Confidence Interval for B | |  |
| --- | --- | --- | --- | --- | --- | --- | --- | --- | --- | --- |
|  | β | p-value | Lower Bound | Upper Bound | VIF | β | p-value | Lower Bound | Upper Bound | VIF |
| **Vaccine Attitudes** | 0.299 | **<.001** | 0.318 | 0.422 | 2.03 | 0.241 | **<.001** | 0.191 | 0.288 | 2.16 |
| **Subjective Norm** | 0.167 | **<.001** | 0.171 | 0.281 | 1.91 | 0.189 | **<.001** | 0.179 | 0.276 | 1.47 |
| **Perceived Severity** | -0.007 | 0.791 | -0.063 | 0.048 | 2.10 | 0.023 | 0.336 | -0.025 | 0.073 | 2.04 |
| **Anticipated Regret** | 0.193 | **<.001** | 0.16 | 0.247 | 1.87 | 0.179 | **<.001** | 0.109 | 0.189 | 2.09 |
| **Vaccine knowledge** | 0.016 | 0.369 | -0.024 | 0.064 | 1.31 | 0.044 | **0.014** | 0.009 | 0.077 | 1.13 |
| **Vaccine benefits** | 0.068 | **<.001** | 0.057 | 0.204 | 1.64 | 0.130 | **<.001** | 0.118 | 0.255 | 2.05 |
| **Vaccine safety** | 0.018 | 0.482 | -0.051 | 0.108 | 2.96 | 0.073 | **0.01** | 0.022 | 0.166 | 2.77 |
| **Trust to Authorities for vaccine approval** | 0.090 | **<.001** | 0.060 | 0.199 | 2.68 | 0.169 | **<.001** | 0.123 | 0.251 | 2.99 |
| **Past influenza vaccine behaviour** | 0.004 | 0.816 | -0.003 | 0.004 | 1.09 | 0.036 | **0.039** | 0.001 | 0.006 | 1.08 |
| **Frequency of past influenza vaccination** | 0.270 | **<.001** | 0.192 | 0.262 | 1.93 | 0.031 | 0.119 | -0.005 | 0.048 | 1.38 |
| **Perceived Control** | -0.019 | 0.257 | -0.070 | 0.019 | 1.24 | 0.009 | 0.626 | -0.027 | 0.045 | 1.23 |
| **Vaccine scepticism** | -0.008 | 0.659 | -0.051 | 0.032 | 1.44 | 0.010 | 0.594 | -0.026 | 0.045 | 1.29 |
| **Have you had coronavirus?** | 0.009 | 0.557 | -0.062 | 0.115 | 1.05 | -0.014 | 0.414 | -0.114 | 0.047 | 1.06 |
| **Have been shielding** | -0.001 | 0.967 | -0.112 | 0.108 | 1.17 | -0.005 | 0.782 | -0.114 | 0.086 | 1.17 |
| **Perceived susceptibility** | 0.041 | 0.053 | -0.001 | 0.099 | 1.91 | -0.023 | 0.303 | -0.064 | 0.02 | 1.70 |
| **Age** | -0.012 | 0.505 | -0.004 | 0.002 | 1.39 | 0.006 | 0.747 | -0.002 | 0.003 | 1.38 |
| **Gender: Female** | 0.012 | 0.430 | -0.049 | 0.114 | 1.06 | -0.013 | 0.460 | -0.103 | 0.046 | 1.08 |
| **Ethnicity: Black/Mixed Black** | 0.038 | **0.014** | 0.042 | 0.380 | 1.07 | 0.025 | 0.152 | -0.041 | 0.265 | 1.08 |
| **General health** | 0.029 | 0.107 | -0.010 | 0.105 | 1.40 | -0.029 | 0.148 | -0.090 | 0.014 | 1.38 |
| **BMI** | 0.026 | 0.107 | -0.001 | 0.014 | 1.12 | 0.005 | 0.776 | -0.006 | 0.008 | 1.12 |
| **Index of Multiple Deprivation** | 0.011 | 0.497 | -0.009 | 0.019 | 1.07 | 0.036 | **0.041** | 0.001 | 0.026 | 1.06 |
| **Healthcare worker status** | -0.038 | **0.014** | -0.183 | -0.021 | 1.05 | -0.034 | 0.053 | -0.146 | 0.001 | 1.05 |

Adjusted R^2^ for influenza vaccine intention: 67.7

Adjusted R^2^ for COVID-19-19 vaccine intention: 59.7

**Table S3.** Multiple linear regression estimates for socio-demographic variables and Influenza and COVID-19 vaccine intention and displaying coefficients [95% CIs] and VIF coefficients (N=1,660)

|  | **Seasonal Influenza vaccine responses** |  | 95% Confidence Interval for B | |  | **COVID-19 vaccine responses** |  | 95% Confidence Interval for B | |  |
| --- | --- | --- | --- | --- | --- | --- | --- | --- | --- | --- |
|  | β | p-value | Lower Bound | Upper Bound | VIF | β | p-value | Lower Bound | Upper Bound | VIF |
| **Age** | **0.196** | <.001 | 0.01 | 0.02 | 1.12 | 0.046 | 0.076 | 0.00 | 0.01 | 1.12 |
| **Gender** (Reference category: males) | **0.065** | 0.008 | 0.05 | 0.30 | 1.03 | -0.015 | 0.536 | -0.14 | 0.07 | 1.03 |
| **Ethnicity** (Reference category: White)  **Black and Mixed Black** | -0.005 | 0.833 | -0.28 | 0.23 | 1.08 | **-0.052** | 0.043 | -0.43 | -0.01 | 1.08 |
| **Asian** | 0.028 | 0.274 | -0.09 | 0.33 | 1.09 | 0.012 | 0.635 | -0.13 | 0.22 | 1.09 |
| **Multiple/Other** | 0.012 | 0.611 | -0.24 | 0.40 | 1.03 | -0.006 | 0.818 | -0.30 | 0.23 | 1.03 |
| **IMD** | **0.057** | 0.019 | 0.00 | 0.05 | 1.03 | **0.068** | 0.006 | 0.01 | 0.04 | 1.03 |
| **HCW** | **0.053** | 0.027 | 0.02 | 0.27 | 1.01 | -0.02 | 0.422 | -0.15 | 0.06 | 1.01 |

Adjusted R^2^ for influenza vaccine intention: 0.043

Adjusted R^2^ for COVID-19-19 vaccine intention: 0.012

**Appendix B**

*Supplementary materials*

**Table S4**. Psychological constructs, measures and their sources

| **Seasonal Influenza/ COVID-19 belief area** | **Factor** | **Psychological theory** | **Statements used to capture** | **Informing study** | **Responses** |
| --- | --- | --- | --- | --- | --- |
| Seasonal influenza COVID-19 vaccine intention | Influenza COVID-19 vaccine intention | Theory of planned behaviour | - When it’s available to me, I will have the flu vaccine - When it’s available to me, I will have a coronavirus vaccine | Sherman et al., 2020 | - Strongly disagree - Disagree - Neither agree nor disagree - Agree - Strongly agree |
| Beliefs regarding vaccination: Influenza/ the COVID-19 pandemic | Perceived severity of influenza/a COVID-19 infection | Health belief model | - Complications from flu/coronavirus would be serious for me - I will be very sick if I get flu/coronavirus | Myers & Goodwin, 2011 | - Strongly disagree - Disagree - Neither agree nor disagree - Agree - Strongly agree |
| Beliefs regarding the Influenza/COVID-19 pandemic | Perceived susceptibility to Influenza/COVID-19 infection | Health belief model | - I believe that I’m at high risk of catching flu/coronavirus compared to others | Myers & Goodwin, 2011 | - Strongly disagree - Disagree - Neither agree nor disagree - Agree - Strongly agree |
| Beliefs regarding the Influenza/COVID-19 pandemic | Trust in the NHS and the UK Government body approving a Influenza/COVID-19 vaccine | N/A | - I believe that a flu/coronavirus vaccine approved by a UK Government body, will be very safe - I believe that a flu/coronavirus vaccine approved by the NHS, will be very safe | Sherman et al., 2020 | - Strongly disagree - Disagree - Neither agree nor disagree - Agree - Strongly agree |
| Beliefs regarding a potential Influenza/COVID-19 vaccine | Influenza/COVID-19 vaccine attitudes | Theory of planned behaviour | - I feel that having a vaccine against flu/coronavirus this year would be: | Myers & Goodwin, 2011 | - Worthless to Valuable - Harmful to Beneficial - Painful to Tolerable |
| Beliefs regarding the influenza/a (potential) COVID-19 vaccine | Influenza/COVID-19 vaccine subjective norms | Theory of planned behaviour | - My family would expect me to be vaccinated for flu/coronavirus - My GP would expect me to be vaccinated for flu/coronavirus - I believe that a flu/coronavirus vaccine approved by the NHS, will be very safe | Myers & Goodwin, 2011 | - Strongly disagree - Disagree - Neither agree nor disagree - Agree - Strongly agree |
| Beliefs regarding the influenza/a (potential) COVID-19 vaccine | Influenza/COVID-19 vaccine anticipated regret | Extended TPB | - Imagine that you caught flu/coronavirus, but that a vaccine might have prevented it - Imagine that you caught flu/coronavirus and passed on to a friend, but that a vaccine might have prevented it - Imagine that you caught flu/coronavirus and passed on to a family member, but that a vaccine might have prevented it | Myers & Goodwin, 2011  Ziarnowski et al., 2009 | - Strongly disagree - Disagree - Neither agree nor disagree - Agree - Strongly agree |
| Beliefs regarding the influenza/a (potential) COVID-19 vaccine | Influenza/COVID-19 vaccine safety | Health belief model/TPB | - I believe that any side-effects from a flu/coronavirus vaccine that I might experience would be mild. - I believe that a flu/coronovirus vaccine that has been fully tested in a clinical trial, will be very safe. | Myers & Goodwin, 2011 | - Strongly disagree - Disagree - Neither agree nor disagree - Agree - Strongly agree |
| Beliefs regarding the influenza/a (potential) COVID-19 vaccine | Influenza/COVID-19 vaccine perceived vaccine knowledge sufficiency | Extended TPB | - I know enough about the safety of a flu/coronavirus vaccine to make an informed decision about whether or not to get vaccinated for coronavirus | Sherman et al., 2020 | - Strongly disagree - Disagree - Neither agree nor disagree - Agree - Strongly agree |
| Beliefs regarding the influenza/a (potential) COVID-19 vaccine | Perceived benefits a mass Influenza/COVID-19 immunisation programme | Health belief model | - If I have a flu/coronavirus vaccine, I’m confident that I will not be able to catch the flu/coronavirus - If I have a flu/coronavirus vaccine, and still caught flu/coronavirus, the severity of my illness will be reduced - If I have a flu/coronavirus vaccine, I won’t be able to spread coronavirus to others - If I have a flu/coronavirus vaccine, I won’t have to socially distance to protect others from flu/coronavirus - Mass flu/coronavirus vaccination, will protect the NHS - Mass flu/coronavirus vaccination, will help the country get back to normal | Myers & Goodwin, 2011  Sherman et al., 2020 | - Strongly disagree - Disagree - Neither agree nor disagree - Agree - Strongly agree |
| Beliefs regarding the influenza/a (potential) COVID-19 vaccine | Influenza/COVID-19 vaccine perceived control | Theory of planned behaviour | - I feel in total control as to whether I will have a flu/coronavirus vaccine | Myers & Goodwin, 2011 | - Strongly disagree - Disagree - Neither agree nor disagree - Agree - Strongly agree |
| Beliefs about vaccines | Vaccine scepticism | Theory of planned behaviour | - I’m someone who is sceptical about all vaccines | Adapted from Sherman et al., 2020 | - Strongly disagree - Disagree - Neither agree nor disagree - Agree - Strongly agree |

**Table S5**. Measures, associations between constructs and scale reliability statistics for influenza vaccine intention

| **Measure** | **Statements used to capture measure** | **Response: 5-point Likert scale** | **Univariate Association with intention (Pearson’s correlation)** | **Scale internal consistency (Cronbach’s alpha/Pearson’s correlation)** |
| --- | --- | --- | --- | --- |
| Influenza vaccine intention | When it’s available to me, I will have a flu vaccine. | Strongly disagree (1) – Strongly agree (5) | - | - |
| Perceived severity of an influenza infection | Complications from flu would be serious for me. | Strongly disagree (1) – Strongly agree (5) | 0.357** | 0.85 |
|  | I will be very sick if I get the flu. |  | 0.251** |  |
|  |  | Items merged- overall | 0.357** |  |
| Perceived susceptibility to influenza infection | I believe that I’m at high risk of catching flu compared to others. | Strongly disagree (1) – Strongly agree (5) | 0.345** | - |
| Trust in the NHS and the UK Government body approving the influenza vaccine | I believe that a flu vaccine approved by a UK Government body, will be very safe. | Strongly disagree (1) – Strongly agree (5) | 0.421** | 0.70 |
|  | I believe that a flu vaccine approved by the NHS, will be very safe. |  | 0.502** |  |
|  |  | Items merged- overall | 0.500** |  |
| Influenza vaccine attitudes | I feel that having a vaccine against the flu this year would be: | Worthless (1) – Valuable (5) | 0.707** | 0.87 |
|  |  | Harmful (1) – Beneficial (5) | 0.664** |  |
|  |  | Painful (1) – Tolerable (5) | 0.465** |  |
|  |  | Items combined-overall | 0.710** |  |
| Influenza vaccine subjective norms | My family would expect me to be vaccinated for flu. | Strongly disagree (1) – Strongly agree (5) | 0.650** | 0.74 |
|  | My GP would expect me to be vaccinated for flu. |  | 0.475** |  |
|  | I will feel under social pressure to be vaccinated for flu. |  | 0.121** |  |
|  |  | Items merged-overall | 0.530** |  |
| Perceived flu vaccine safety | I believe that any side-effects from a flu vaccine that I might experience would be mild. | Strongly disagree (1) – Strongly agree (5) | 0.448** | 0.81 |
|  | I believe that a flu vaccine that has been fully tested in a clinical trial, will be very safe. |  | 0.468** |  |
|  |  | Items merged- overall | 0.500** |  |
| Influenza vaccine anticipated regret | Imagine that you caught the flu, but that a vaccine might have prevented it. | Not at all (1) – A great deal (5) | 0.641** | 0.94 |
|  | Imagine that you caught the flu and passed on to a friend, but that a vaccine might have prevented it. |  | 0.598** |  |
|  | Imagine that you caught the flu and passed on to a family member, but that a vaccine might have prevented it. |  | 0.575** |  |
|  |  | Items merged-overall | 0.639** |  |
| (Perceived) influenza vaccine knowledge sufficiency | I know enough about the safety of a flu vaccine to make an informed decision about whether or not to get vaccinated for flu. | Strongly disagree (1) – Strongly agree (5) | 0.286** | - |
| Perceived benefits of a mass influenza immunisation programme | If I have a flu vaccine, I’m confident that I will not be able to catch the flu. | Strongly disagree (1) – Strongly agree (5) | 0.382** | 0.79 |
|  | If I have a flu vaccine, and still caught the flu, the severity of my illness will be reduced. |  | 0.435** |  |
|  | If I have a flu vaccine, I won’t be able to spread the flu to others. |  | 0.219** |  |
|  | If I have a flu vaccine, I won’t have to socially distance to protect others from the flu. |  | 0.04** |  |
|  | Mass flu vaccination, will protect the NHS. |  | 0.542** |  |
|  | Mass flu vaccination, will help the country get back to normal. |  | 0.448** |  |
|  |  | Items merged-overall | 0.510** |  |
| Influenza vaccine perceived control | I feel in total control as to whether I will have a flu vaccine. | Strongly disagree (1) – Strongly agree (5) | 0.170** | - |
| Vaccine scepticism | Vaccine scepticism | I’m someone who is sceptical about all vaccines | Strongly disagree (1) – Strongly agree (5) | -.270** |

* p < 0.05, ** p < 0.01, *** p < 0.001

**Table S6**. Measures, associations between constructs and scale reliability statistics for COVID-19 vaccine intention

| **Measure** | | **Statements used to capture measure** | **Response: 5-point Likert scale** | | **Univariate Association with intention (Pearson’s correlation)** | | **Scale internal consistency (Cronbach’s alpha/Pearson’s correlation)** | |
| --- | --- | --- | --- | --- | --- | --- | --- | --- |
| COVID-19 vaccine intention | | When it’s available to me, I will have a coronavirus vaccine. | Strongly disagree (1) – Strongly agree (5) | | - | | - | |
| Perceived severity of a COVID-19 infection | | Complications from coronavirus would be serious for me. | Strongly disagree (1) – Strongly agree (5) | | 0.240** | | 0.81 | |
|  | | I will be very sick if I get coronavirus. |  | | 0.235** | |  | |
|  | |  | Items merged - overall | | 0.249** | |  | |
| Perceived susceptibility to COVID-19 infection | | I believe that I’m at high risk of catching coronavirus compared to others. | Strongly disagree (1) – Strongly agree (5) | | 0.175** | | - | |
| Trust in the NHS and the UK Government body approving a COVID-19 vaccine | | I believe that a coronavirus vaccine approved by a UK Government body, will be very safe. | Strongly disagree (1) – Strongly agree (5) | | 0.556** | | 0.71 | |
|  | | I believe that a coronavirus vaccine approved by the NHS, will be very safe. |  | | 0.614** | |  | |
|  | |  | Items merged- overall | | 0.634** | |  | |
| COVID-19 vaccine attitudes | | I feel that having a vaccine against coronavirus this year would be: | Worthless (1) – Valuable (5) | | 0.671** | | 0.90 | |
|  | |  | Harmful (1) – Beneficial (5) | | 0.648** | |  | |
|  | |  | Painful (1) – Tolerable (5) | | 0.450** | |  | |
|  | |  | Items merged - overall | | 0.650** | |  | |
| COVID-19 vaccine subjective norms | | My family would expect me to be vaccinated for coronavirus. | Strongly disagree (1) – Strongly agree (5) | | 0.610** | | 0.74 | |
|  | | My GP would expect me to be vaccinated for coronavirus. |  | | 0.365** | |  | |
|  | | I will feel under social pressure to be vaccinated for coronavirus. |  | | 0.282** | |  | |
|  | |  | Items merged- overall | | 0.515** | |  | |
| Perceived COVID-19 vaccine safety | I believe that any side-effects from a coronovirus vaccine that I might experience would be mild. | | | Strongly disagree (1) – Strongly agree (5) | | 0.468** | | 0.81 |
|  | I believe that a coronovirus vaccine that has been fully tested in a clinical trial, will be very safe. | | |  | | 0.572** | |  |
|  |  | | | Items merged- overall | | 0.579** | |  |
| COVID-19 vaccine anticipated regret | | Imagine that you caught coronavirus, but that a vaccine might have prevented it. | Not at all (1) – A great deal (5) | | 0.671** | | 0.95 | |
|  | | Imagine that you caught coronavirus and passed on to a friend, but that a vaccine might have prevented it. |  | | 0.648** | |  | |
|  | | Imagine that you caught coronavirus and passed on to a family member, but that a vaccine might have prevented it. |  | | 0.450** | |  | |
| (Perceived) COVID-19 vaccine knowledge sufficiency | | I know enough about the safety of a coronavirus vaccine to make an informed decision about whether or not to get vaccinated for coronavirus. | Strongly disagree (1) – Strongly agree (5) | | 0.089** | | - | |
| Perceived benefits a mass COVID-19 immunisation programme | | If I have a coronavirus vaccine, I’m confident that I will not be able to catch the coronavirus. | Strongly disagree (1) – Strongly agree (5) | | 0.449** | | 0.85 | |
|  | | If I have a coronavirus vaccine, and still caught coronavirus, the severity of my illness will be reduced. |  | | 0.483** | |  | |
|  | | If I have a coronavirus vaccine, I won’t be able to spread coronavirus to others. |  | | 0.337** | |  | |
|  | | If I have a coronavirus vaccine, I won’t have to socially distance to protect others from coronavirus. |  | | 0.208** | |  | |
|  | | Mass coronavirus vaccination, will protect the NHS. |  | | 0.613** | |  | |
|  | | Mass coronavirus vaccination, will help the country get back to normal. |  | | 0.604** | |  | |
|  | |  | Items merged- overall | | 0.603** | |  | |
| COVID-19 vaccine perceived control | | I feel in total control as to whether I will have a coronavirus vaccine. | Strongly disagree (1) – Strongly agree (5) | | 0.190** | | - | |
| Vaccine scepticism | | I’m someone who is sceptical about all vaccines | Strongly disagree (1) – Strongly agree (5) | | -.303** | | - | |

* p < 0.05, ** p < 0.01, *** p < 0.001
